# Anaerozeibacter quisquiliarum gen. nov., sp. nov., a novel mesophilic bacterium isolated from a laboratory-scale methanogenic landfill bioreactor digesting maize, and proposal of Anaerozeibacteraceae fam. nov., representing a new family within the order Eubacteriales

**DOI:** 10.1099/ijsem.0.006985

**Published:** 2025-12-10

**Authors:** Abdelaziz El Houari, Morgan Carpenter, Daniel Chaplin, Peter Golyshin, James E. McDonald

**Affiliations:** 1 School of Environmental and Natural Sciences, Environment Centre Wales, Bangor University, Bangor, UK; 2 Centre for Environmental Biotechnology (CEB), Bangor University, Bangor, UK; 3 Institute of Microbiology and Infection, Birmingham Institute for Forest Research, School of Biosciences, University of Birmingham, Birmingham, UK

**Keywords:** *Eubacteriales*, laboratory-scale bioreactor, landfill, maize feedstock, methanogens enrichment

## Abstract

Bacteria involved in the anaerobic degradation of lignocellulosic waste in landfill sites play crucial roles in carbon turnover and biogas generation. In this study, we isolated and characterized a novel anaerobic bacterium, strain meth-B3ᵀ, from a laboratory-scale methanogenic bioreactor fed with maize-based biomass. Cells were Gram-stain-negative, non-spore-forming, motile rods with optimal growth at 35 °C, pH 7.0 and 0.7% sodium chloride (NaCl). Strain meth-B3ᵀ utilized a broad spectrum of carbohydrates, amino acids and organic acids, including glucose, cellobiose, glycerol, sucrose, maltose and various nitrogenous compounds. It fermented glucose into acetate, butyrate, lactate, propionate, valerate and ethanol. Whole-genome sequencing revealed a 3.8 Mbp genome with a G+C content of 62.65 mol%. Phylogenomic analyses based on 16S rRNA and conserved marker genes placed strain meth-B3ᵀ within the order *Eubacteriales*, forming a distinct clade from other known families. Comparative genomic metrics (average nucleotide identity, ≤69.4%; average amino acid identity, ≤54.2%; percentage of conserved protein, ≤35.2%) confirmed that strain meth-B3ᵀ represents a novel genus and family. Notably, carbohydrate-active enzyme and Clusters of Orthologous Groups (COG) functional profiling revealed an extensive suite of enzymes with potential activities against cellulose, xylan, starch and other maize-derived polymers, underscoring its ecological and biotechnological relevance in biomass degradation and biogas production. On the basis of genotypic and phenotypic distinctions, we propose the name *Anaerozeibacter quisquiliarum* gen. nov., sp. nov., with strain meth-B3ᵀ (=DSM 112769ᵀ=ATCC TSD-269ᵀ) being the type strain, and designate *Anaerozeibacteraceae* fam. nov. within the order *Eubacteriales* to accommodate this lineage.

## Data summary

Supplementary material can be found at https://doi.org/10.6084/m9.figshare.30445061.v1 [1].

## Introduction

Landfill sites and anaerobic digesters are engineered ecosystems designed for waste decomposition and energy recovery through the microbial production of biogas, comprising mainly methane and carbon dioxide [2,3]. A significant fraction of this biodegradable input comprises lignocellulosic biomass, such as maize silage, food residues and agricultural waste, which accumulates in landfills or is directly fed into anaerobic digesters [4,5]. The conversion of these complex substrates into biogas is driven by tightly coordinated microbial consortia, whose metabolic activities underpin both the hydrolytic breakdown of polymers like cellulose and hemicellulose, and the subsequent fermentation and methanogenesis stages [6,8].

Micro-organisms from the phylum *Bacillota*, including those affiliated with the order *Eubacteriales*, play a critical ecological role in these environments [9,10]. Members of this order are often specialized in fermentative metabolism, hydrogen and short-chain fatty acid production, and the degradation of recalcitrant plant material [11,14]. At the time of writing, the order *Eubacteriales* encompasses a wide diversity of anaerobic taxa, many of which remain poorly characterized in terms of their ecological roles and phylogenetic relationships [15]. Among a total of 56 distinct family-level taxa, of which 38 have been validly published under the rules of the International Code of Nomenclature of Prokaryotes, 18 are provisionally named or await formal validation. The non-validly published taxa included in this study are indicated in quotation marks and were retained to provide a comprehensive assessment of genomic diversity and taxonomic relationships within the order *Eubacteriales* (https://lpsn.dsmz.de/order/eubacteriales).

In this study, we describe the isolation and characterization of a novel bacterium, designated strain meth-B3^T^, recovered from a laboratory-scale methanogenic bioreactor fed with maize biomass. This reactor was inoculated with microbial consortia derived from landfill leachate. Phylogenetic analyses based on 16S rRNA gene sequences and genome-wide comparisons indicate that strain meth-B3^T^ does not affiliate with any existing family within *Eubacteriales*. Instead, its distinct genetic and phenotypic features support the proposal of a new genus, *Anaerozeibacter* gen. nov., and species, *Anaerozeibacter quisquiliarum* sp. nov., for which meth-B3^T^ is the type strain. Furthermore, the data justify the establishment of a novel family, *Anaerozeibacteraceae* fam. nov., to accommodate this lineage.

## Source of micro-organisms and isolation

To establish a representative microbial community from municipal solid waste environments, inocula were prepared using mixtures of landfill leachate and drilled solid waste, as previously described [11,16]. Briefly, drilled waste and leachate samples were collected from the Ruabon and Hafod landfill sites (UK), respectively, handled anaerobically and used to inoculate laboratory-scale synthetic landfill microbiome (SLM) stirred bioreactors. Reactors were supplemented with 2% (w/v) maize as the primary feedstock and operated under mesophilic conditions (35 °C, 150 r.p.m.) for 28 days. At peak biogas production (recorded at day 21), sludge was sampled and used to initiate methanogenic enrichment cultures in basal medium [17] supplemented with formate, methanol, methylamine and acetate (10 mM each), yeast extract (2 g l⁻¹) and penicillin (0.5 g l⁻¹), under a N_₂_/H_₂_/CO_₂_ (80:10:10) atmosphere at 1 bar.

The basal medium used for enrichment of methanogenic microbiota consisted of mineral salts and trace elements [18] modified by Imhoff-Stuckle and Pfennig [19], resazurin (0.1% w/v), bicarbonate, sulphide and vitamins [20], with a final pH adjusted to 7.1. Medium preparation and all anaerobic manipulations were performed under strict anoxic conditions using O_₂_-free N_₂_ gas, and medium was sterilized by autoclaving at 121 °C for 20 min. Enrichments were incubated at 35 °C for 4 weeks, and growth was assessed via methane and carbon dioxide production. Community dynamics of both the SLM bioreactors and the enrichment cultures were monitored by 16S rRNA gene profiling using the Illumina MiSeq platform (data not shown).

Following three sequential subcultures under identical conditions, isolation was conducted using high-throughput dilution-to-extinction in 96-well microplates, as previously described [11,16]. Briefly, optimal dilutions were determined empirically to ensure that 50% of wells exhibited positive growth. Microplates were sealed with gas-permeable film and incubated under anaerobic conditions with N_₂_/H_₂_/CO_₂_ (80:10:10) within a Whitley DG250 Anaerobic Workstation. Two morphologically similar isolates (meth-B3^T^ and meth-H3) were recovered from the highest positive dilution (10⁻^7^) and subjected to purity checks via microscopy and growth on solid medium. 16S rRNA gene sequencing confirmed both isolates to be identical (100% sequence identity). For the present study, strain meth-B3^T^ was selected for full characterization.

## Morphological and echo-physiological characterization

**Fig. 1.**
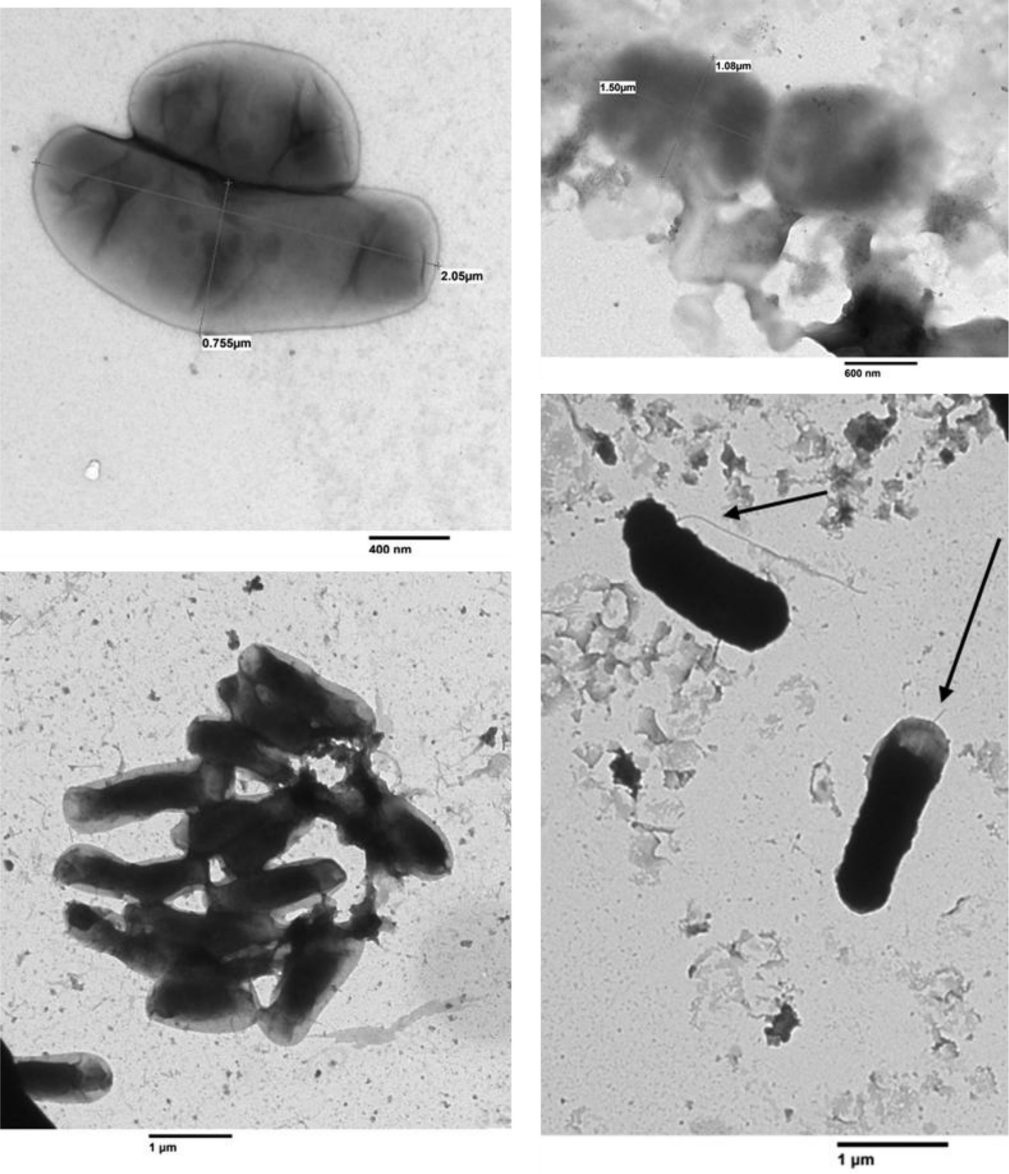
Transmission electron micrographs of strain meth-B3^T^ cells, grown in FAB medium, forming a monotrichous flagellum (bar, 1 µm).

Strain meth-B3^T^ exhibited optimal growth in basal medium supplemented with 30 g l⁻¹ Fastidious Anaerobic Broth (FAB; Neogen Culture Media, NCM0199C), hereafter referred to as modified FAB medium. Unless otherwise indicated, this medium was routinely used for cultivation. Morphological and cellular characteristics and sporulation were examined using phase-contrast microscopy (Zeiss Axioplan 2 Fluorescence microscope, FluoArc 100). Cell shape, dimensions and flagella were identified by transmission electron microscopy (TEM) performed at the Animal and Plant Health Agency (APHA; http://apha.defra.gov.uk/apha-scientific/index.htm). Gram-staining was conducted using conventional methods and further validated with the NaOH-based procedure described by Wada *et al*. [21]. Cells of strain meth-B3^T^ were rod-shaped, occasionally forming pairs or small aggregates, and exhibited active motility. Cell dimensions varied, measuring ~0.75–1.08 µm in width and 1.50–2.08 µm in length, as observed by TEM (Fig. 1). Cells stained Gram-negative, and TEM analysis confirmed the presence of polar flagella, consistent with the motility observed under phase-contrast microscopy. This motile phenotype distinguishes meth-B3^T^ from its closest phylogenetic relatives, including ‘*Beduinella massiliensis*’ Marseille-P2846^T^ [22], *Aristaeella hokkaidonensis* R-7^T^ and *Aristaeella lactis* WTE2008^T^ [23], *Luoshenia tenuis* NSJ-44^T^ [15] and *Christensenella intestinihominis* AF73-05CM02^T^ [24], all of which are non-motile. When grown on modified FAB agar medium, strain meth-B3^T^ formed cream, spherical colonies ~1–2 mm in diameter after 1 week of incubation.

The aerotolerance of strain meth-B3^T^ was evaluated by incubating cultures in both liquid and solid media prepared without HCl-cysteine or Na_₂_S·9 H_₂_O, and exposed to atmospheric oxygen. No growth was observed under these aerobic conditions, indicating that strain meth-B3^T^ is a strict anaerobe. The presence of endospores in strain meth-B3^T^ was assessed by phase-contrast microscopy and further tested using a heat-resistance (sporulation) assay [11,16]. Exponentially growing cells were subjected to thermal shock following cold incubation (5 min on ice), then exposed to 80 °C for 10 or 20 min, and to 90 °C for 10 min. Following heat treatment, cells were transferred to fresh anaerobic medium and incubated under optimal growth conditions. No regrowth was detected after 1 week, and microscopy revealed no evidence of spores or heat-resistant structures. Positive and negative controls (sporulating and non-sporulating reference strains) confirmed the validity of the test. The inability of meth-B3^T^ to form spores is consistent with its closest phylogenetic relatives, including *‘B. massiliensis’* Marseille-P2846^T^, *A. hokkaidonensis* R-7^T^ and *A. lactis* WTE2008^T^, *L. tenuis* NSJ-44^T^ and *C. intestinihominis* AF73-05CM02^T^, all of which are non-sporulating [15,22,24]. In contrast to several thermophilic spore-forming clostridial species [13,25, 26], meth-B3^T^ was isolated from a mesophilic anaerobic environment and does not exhibit traits associated with spore formation.

## Identification, phylogenetic analyses and genomic features

Genomic DNA from strain meth-B3^T^ was extracted using the DNeasy UltraClean Microbial Kit (Qiagen), following the manufacturer’s instructions. A 1.8 ml aliquot of exponentially growing culture was centrifuged, and the resulting pellet was processed for DNA isolation. Elution was performed in nuclease-free elution buffer (EB; 10 mM Tris-HCl, pH 8.0). DNA integrity was assessed by electrophoresis on a 1% (w/v) agarose gel using a 1 kb molecular weight ladder as a reference. DNA concentration was measured with the Qubit™ dsDNA HS Assay Kit on a Qubit™ 3 Fluorometer (Invitrogen, Thermo Fisher Scientific). For 16S rRNA gene amplification, 2 µl of genomic DNA was used as a template in a 50 µl PCR reaction volume containing 1× MyTaq™ Red Mix (Meridian Bioscience) and primers 27F (5′-TGAGCCATGATCAAACTCT-3′) and 1492R (5′-GGWTACCTTGTTACG-3′) [27], each at a final concentration of 0.8 µM. PCR was conducted with an initial denaturation step at 94 °C for 5 min, followed by 35 cycles of denaturation (94 °C for 45 s), annealing (55 °C for 45 s) and extension (72 °C for 90 s), with a final elongation at 72 °C for 10 min. Amplicons were purified using the QIAquick PCR Purification Kit (Qiagen) and submitted for Sanger sequencing at GENEWIZ (Takeley, UK). The resulting 16S rRNA gene sequence was quality-checked, trimmed and compared against reference sequences in the NCBI (National Center for Biotechnology Information) GenBank database using the BLASTn algorithm (http://www.ncbi.nlm.nih.gov/BLAST/). The closest 16S rRNA gene sequence matches in GenBank for strain meth-B3^T^ were uncultured bacterial clones, each showing ≤95.77% identity, indicating significant phylogenetic divergence. The top hit was clone RL183_aao01d12 (95.77% identity; GenBank accession no. DQ800642.1), obtained from human faeces. The second closest match was clone MgMjR-026 (95.34% identity; AB234483.1), derived from the gut of termites, followed by clone PeHg22 (95.09% identity; FJ374207.1), isolated from a larval hindgut. These findings further support the novelty of strain meth-B3^T^ and suggest its affiliation with an as-yet uncharacterized lineage within the order *Eubacteriales*.

**Fig. 2.**
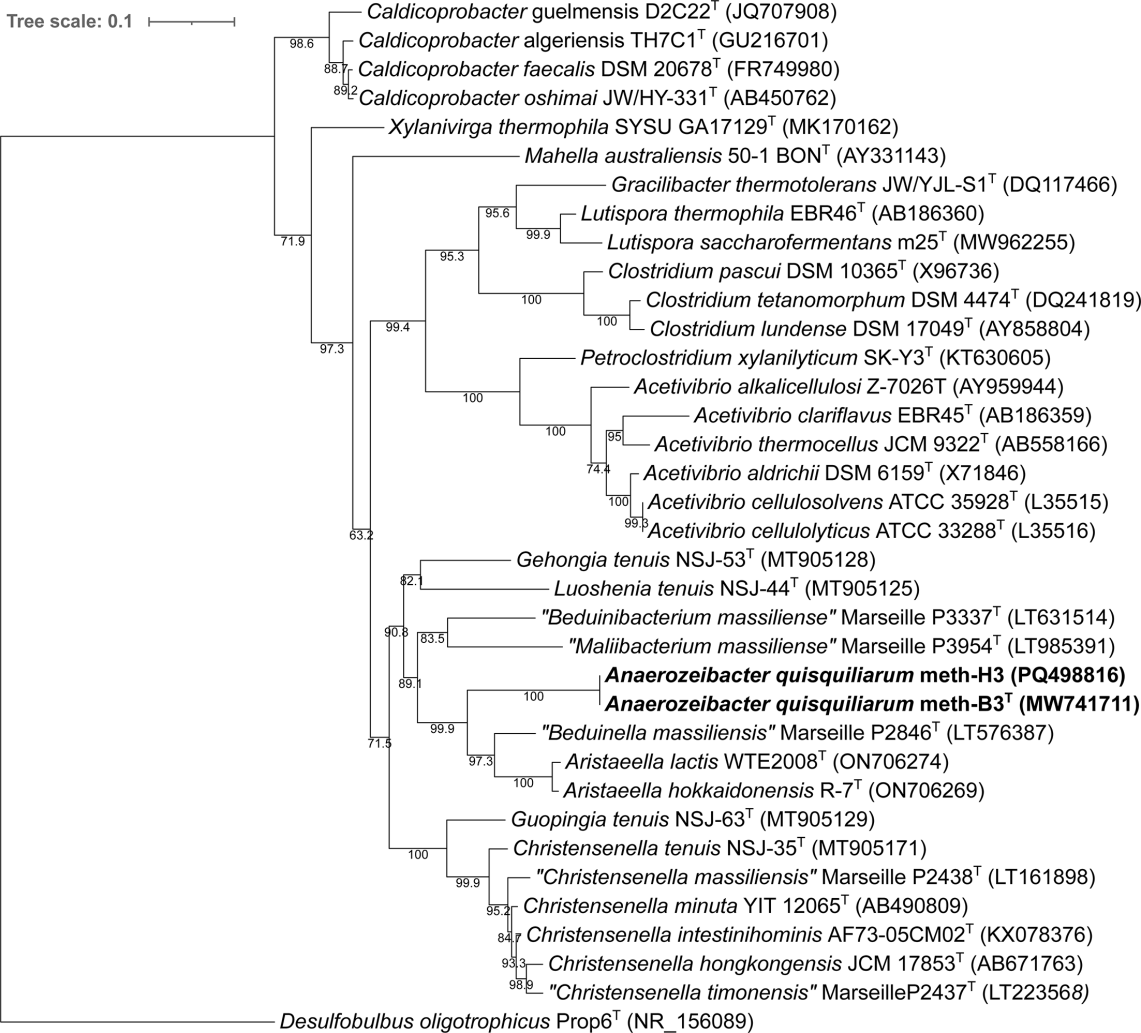
Phylogenetic evolutionary dendrogram constructed by the ML method showing the affiliation of *A. quisquiliarum* (both strains meth-B3^T^ and meth-H3) 16S rRNA gene sequences (1,454 bp) to their closest relative reference sequences obtained from the NCBI GenBank database (accession numbers in brackets). *Desulfobulbus oligotrophicus* Prop6^T^ (NR_156089) was used as an outgroup to root the tree. Bootstrap values are represented at nodes. The bar represents 0.1 substitutions per site. The isolated strains are shown in bold.

The phylogenetic relationships between the 16S rRNA gene sequences of both strains meth-B3^T^ and meth-H3 and their closest related species among the *Eubacteriales* were inferred using a two-step approach. Initially, all sequences were aligned with Clustal Omega (v1.2.4) [28], ensuring accurate alignment of homologous regions across the dataset. The resulting multiple sequence alignment was subsequently analysed using IQ-TREE (v2.4.0) [29] to construct a maximum-likelihood (ML) phylogenetic tree. The GTR+G nucleotide substitution model (General Time Reversible with Gamma-distributed rate variation among sites) was applied, and branch support was evaluated through 1,000 ultrafast bootstrap replicates. The final tree was visualized using iTOL (Interactive Tree of Life) [30], enabling detailed inspection of phylogenetic structure and annotation of support values. Additionally, pairwise estimates of evolutionary divergence between the meth-B3^T^ and meth-H3 strains and their closest phylogenetic neighbours were calculated. This analysis was performed using the Maximum Composite Likelihood method [31] and conducted in mega11 software [32]. Ambiguous positions were eliminated by pairwise deletion, resulting in a final dataset comprising 1,454 aligned positions across 36 nucleotide sequences. These estimates provided a quantitative measure of sequence divergence and complemented the tree-based approach in evaluating taxonomic relatedness (Table S1, available in the online Supplementary Material). Phylogenetic analysis based on full-length 16S rRNA gene sequences revealed that both strains meth-B3^T^ and meth-H3 are identical, sharing 100% sequence similarity, and together represent a single novel taxon for which the name *A. quisquiliarum* is proposed (Fig. 2). The closest related species was *‘B. massiliensis’* Marseille P2846^T^ (LT576387), with a 16S rRNA gene sequence similarity of 88.29% [22]. Additional phylogenetic neighbours included *A. hokkaidonensis* R-7^T^ (ON706269) and *A. lactis* WTE2008^T^ (ON706274), with 86.82% and 86.80% similarity, respectively [23]. Lower levels of sequence identity were observed with *L. tenuis* NSJ-44^T^ (MT905125) [15], *C. intestinihominis* AF73-05CM02^T^ [24] and *Gehongia tenuis* NSJ-53^T^ (MT905128) [15], showing similarities ranging from 83.74% to 84.86%. These values are well below the accepted genus-level threshold of 94.5% and the family-level threshold of ~90% for 16S rRNA gene sequence identity [33,34], providing strong evidence that strain meth-B3^T^ represents not only a novel species and genus but also likely a distinct lineage at the family level within the *Eubacteriales*.

To support the phylogenetic and taxonomic findings, the genome of strain meth-B3^T^ was sequenced. Genomic DNA extracted for 16S rRNA gene amplification was used for library preparation and sequenced on the Illumina NovaSeq platform with 2×250 bp paired-end reads (MicrobesNG; Birmingham Research Park, UK). Raw reads were quality-trimmed using Trimmomatic v0.30 [35] and assembled *de novo* with SPAdes v3.12 [36]. Contigs shorter than 1,000 bp were removed, and genome annotation was performed using Prokka v1.14 [37]. The final draft genome, deposited in GenBank under accession number JBISDI000000000, has a length of 3,827,363 bp from 86 contigs. The draft genome has an N50 contig length of 75,459 bp. There were 3,505 protein-coding genes, 45 tRNAs, 1 transfer-messenger RNA (tmRNA) and 3 rRNA gene copies predicted (File S1). CheckM analysis showed that the assembled genome completeness of strain meth-B3^T^ was 98.76%. The G+C content was 62.65 mol%.

Comparative genomic analysis (File S2) revealed that the G+C content of strain meth-B3ᵀ (62.65%) is markedly higher than that of several phylogenetically related taxa (Fig. 2). For example, *‘B. massiliensis’* (60.75%) and ‘*Maliibacterium massiliense’* (60.82%) have moderate differences in G+C content (1.88% and 1.83%, respectively), while more distant relatives such as *A. hokkaidonensis* (53.04%) and *A. lactis* (53.5%) differ substantially, by 9.61% and 9.15%, respectively. This trend is even more pronounced when compared with typical members of the *Clostridiaceae*, such as *Clostridium lundense* (29.28%) and *Clostridium tetanomorphum* (29.13%), which differ by over 30%. This clear divergence in G+C content, alongside distinct phylogenetic placement, supports the taxonomic distinction of *strain* meth-B3ᵀ as a novel lineage. Digital DNA–DNA hybridization (dDDH) analysis was performed using the Genome-to-Genome Distance Calculator (GGDC v3.0; http://ggdc.dsmz.de/distcalc2.php), applying the recommended formula d0, which calculates the length of all heat shock proteins divided by total genome length [38]. The highest genomic similarity of strain meth-B3ᵀ was observed with *‘B. massiliensis’* Marseille-P2846ᵀ (13.0%), followed by ‘*M. massiliense’* Marseille-P3954ᵀ (12.8%), *A. hokkaidonensis* R-7ᵀ (12.7%) and *A. lactis* WTE2008ᵀ (12.6%). All remaining type strains, including those representing the genera *Christensenella*, *Lutispora*, *Luoshenia*, *Gehongia* and *Xylanivirga*, exhibited dDDH values ranging from 12.5% to 12.6%. These values are substantially below the accepted species delineation threshold of 70% [39,42], clearly indicating that meth-B3ᵀ does not belong to any previously described species. The consistently low genome-to-genome relatedness across all reference taxa further reinforces the distinctiveness of strain meth-B3ᵀ and supports its assignment to a novel lineage within the order *Eubacteriales*.

**Fig. 3.**
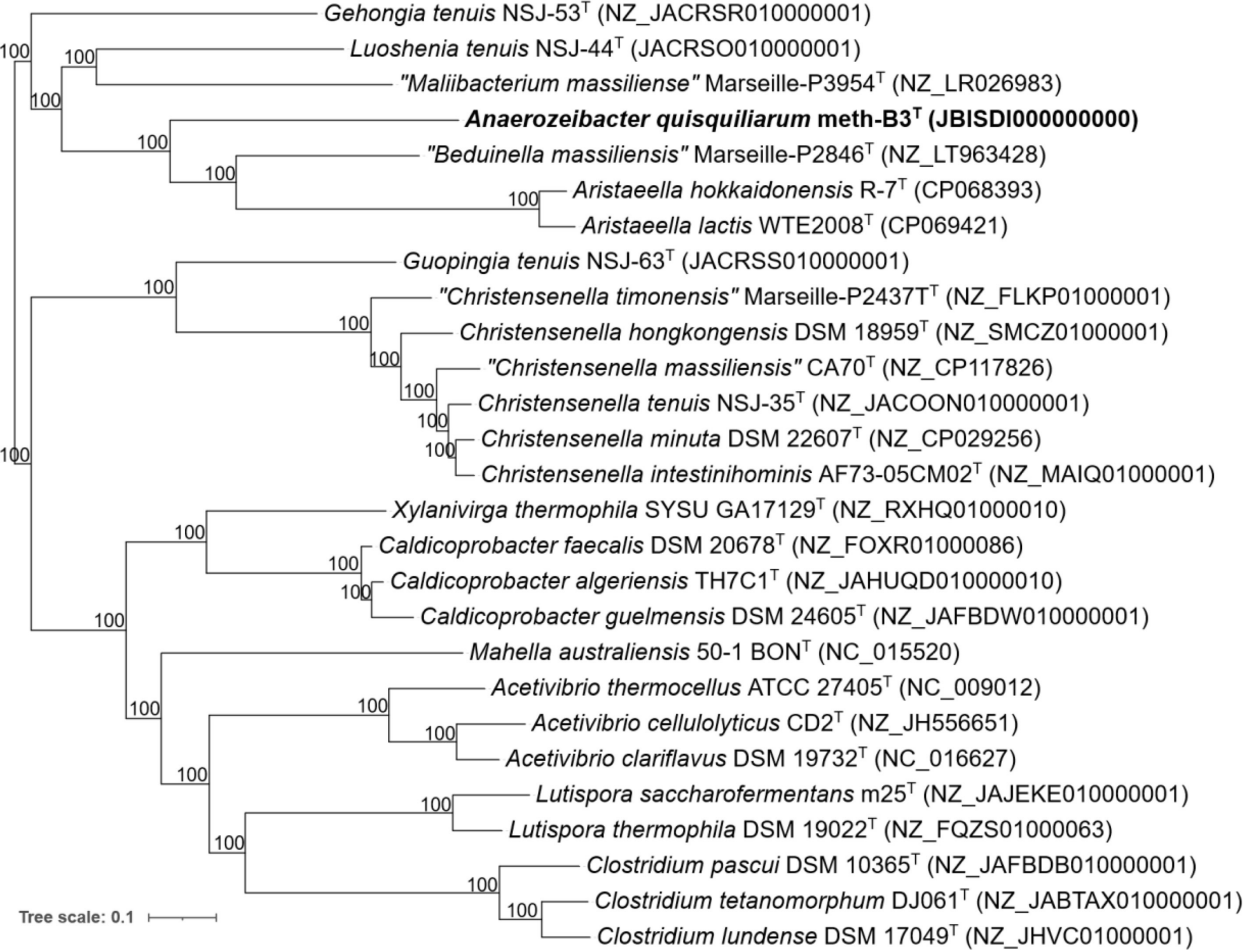
Whole-genome-based phylogenetic tree showing the affiliation of the genome sequence of strain meth-B3^T^ to publicly available reference genomes of its closest relatives (GenBank accession numbers in brackets). The genomes were aligned using the GTDB-Tk toolkit, and the phylogenetic tree was constructed using RAxML-NG. Bootstrap values are represented at nodes. The bar represents 0.1 substitutions per site. The isolated strain meth-B3^T^ (present study) is shown in bold.

Genome-based comparisons were performed to evaluate the phylogenetic placement of strain meth-B3ᵀ relative to publicly available genomes of related taxa. Phylogenomic reconstruction, carried out using GTDB-Tk v2.1.1 [43] for marker gene extraction and RAxML-NG v0.9.0 [44] for ML inference, yielded a tree topology consistent with the 16S rRNA gene-based phylogeny (Fig. 2; Table S1). Strain meth-B3ᵀ formed a distinct monophyletic clade and was clearly separated from all other closely related genomes (Fig. 3). Its closest phylogenomic neighbours included ‘*M. massiliense’* Marseille-P3954ᵀ, *‘B. massiliensis’* Marseille-P2846ᵀ and *Aristaeella* species (*A. hokkaidonensis* R-7ᵀ and *A. lactis* WTE2008ᵀ), yet it remained genomically divergent from these taxa. This genome-based analysis further reinforces the novel taxonomic status of strain meth-B3ᵀ and its assignment to a new genus within the *Eubacteriales*.

To assess genome-level relatedness and taxonomic placement of strain meth-B3^T^, average nucleotide identity (ANI) and average amino acid identity (AAI) were calculated against publicly available genomes from phylogenetically related taxa closely related to strain meth-B3^T^. ANI values were obtained using pyani v0.2.8 (https://github.com/widdowquinn/pyani), while AAI was computed using CompareM v0.1.2 (https://github.com/dparks1134/CompareM). Additionally, a percentage of conserved protein (POCP) analysis was performed using the POCP-nf pipeline (https://github.com/hoelzer/pocp), a Nextflow-based tool for automated POCP calculation [45]. The highest ANI values were observed with *C. tetanomorphum* DJ061ᵀ (69.41%), *‘B. massiliensis’* Marseille-P2846ᵀ (67.72%) and ‘*M. massiliense’* Marseille-P3954ᵀ (66.69%) (File S3). All ANI values fell well below the ≥95% species and ~83–85% genus thresholds typically used for taxonomic delineation [46,49]. Notably, meth-B3ᵀ exhibited a maximum ANI of only 69.41%, which is below the proposed ~75–77% ANI boundary that distinguishes bacterial families [50,51]. These results, taken together with phylogenomic analyses and dDDH values far below the 70% species cutoff, support the classification of *strain* meth-B3ᵀ as representing not only a novel genus but also a distinct family within the order *Eubacteriales*. AAI comparisons (File S4) supported this finding, with all values falling between 47.8% and 54.2%. The highest AAI was observed with *‘B. massiliensis’* Marseille-P2846ᵀ (54.25%), followed by members of the genera *Aristaeella*, *Luoshenia* and ‘*Maliibacterium’*, all ranging from 52.1% to 52.49%. These values fall well below the established genus boundary of ~65–72% and significantly under the proposed family-level AAI threshold of ~60–65% [50,52,54]. POCP values between strain meth-B3ᵀ and its phylogenetically closest type strains (File S5) ranged from 21.64% to 35.16%, with the highest POCP observed with *‘B. massiliensis’* Marseille-P2846ᵀ (35.16%). All values fall below the 50% threshold proposed for genus-level boundaries and are markedly lower than the ≥55% typically considered indicative of a shared family affiliation [54,55]. This pronounced phylogenomic divergence, in conjunction with 16S rRNA gene sequence divergence, and coupled with low AAI and ANI values and the lack of close genomic similarity, supports the assertion that strain meth-B3^T^ represents not only a novel species and genus but also supports the proposal of a new family, *Anaerozeibacteraceae* fam. nov., within the order *Eubacteriales*.

## Physiological, biochemical, chemotaxonomic and functional genomic characterization

The physiological growth parameters of strain meth-B3ᵀ were determined under strictly anaerobic conditions using FAB medium (10 ml final volume) distributed in 20 ml headspace vials (QUMA) sealed with butyl rubber septa and aluminium crimp caps. Growth temperature was tested across a range from 4 to 60 °C, with the standard pH maintained at 7.1 unless otherwise specified. For pH optimization, medium was buffered with 10 mM MES (pH 4.0–6.0), HEPES (pH 6.0–8.0) or CHES (pH 8.0–10.0), and adjusted using sterile anaerobic 1 M HCl or NaOH. NaCl tolerance was assessed at concentrations ranging from 0% to 10% (w/v), with salts added directly prior to autoclaving. Growth was monitored spectrophotometrically by measuring optical density at 600 nm using a Jenway 7300 Visible Spectrophotometer. Eco-physiological characterization of strain meth-B3ᵀ revealed a distinct mesophilic growth profile, with an optimal growth temperature of 35 °C and a temperature growth range between 25 and 37 °C. This contrasts with its closest phylogenetic neighbours, including *A. hokkaidonensis* R-7ᵀ and *A. lactis* WTE2008ᵀ, both of which demonstrate broader temperature ranges (30–45 °C), indicative of a more thermotolerant physiology [23]. The optimal pH for meth-B3ᵀ was 7.0, with growth occurring between pH 6.5 and 8.0. This range aligns with other *Christensenellaceae*-related strains, such as *C. intestinihominis* AF73-05CM02ᵀ (optimum pH 6.5–7.0) and *Christensenella minuta* YIT 12065ᵀ (optimum pH 7.5), yet it is slightly narrower in scope [15,24, 56,58]. Salt tolerance experiments showed that meth-B3ᵀ is slightly NaCl-tolerant, with growth up to 0.8% NaCl (optimum 0.7%), whereas *Aristaeella* species grow only in the presence of 0.5% NaCl [23], and *C. minuta* is tolerant of up to 5% NaCl [58], further supporting its genomic distinctiveness. Together, these phenotypic traits, particularly its mesophilic preference, salt tolerance, motility and elevated G+C content, clearly delineate strain meth-B3ᵀ from previously described taxa within the order *Eubacteriales*.

Substrate utilization was investigated in triplicate using exponentially growing cells resuspended in pre-reduced basal medium without l-cysteine hydrochloride anhydrous, in both the absence and the presence of 0.2% (w/v) yeast extract under N_2_ as the gas phase. Substrate utilization was determined for the following substrates (20 mM each): sugars, amino acids, organic acids and alcohols. Furthermore, the strain was tested for its capacity to utilize complex substrates (1% each) such as yeast extract, tryptone, peptone, casamino acids, starch, crystalline cellulose (Avicel), xylan and casein. The key phenotypic and metabolic traits that differentiate strain meth-B3ᵀ from its closest phylogenetic relatives within the order *Eubacteriales* are summarized in Table 1. Strain meth-B3ᵀ did not require yeast extract for growth, but its presence enhanced its growth. Strain meth-B3ᵀ was able to grow in the absence of yeast extract on a wide range of substrates, including glucose, fructose, galactose, lactose, maltose, cellobiose, sucrose, mannitol, arabinose, raffinose, glycine, isoleucine, succinate, malate and gallic acid. However, in the presence of yeast extract, strain meth-B3ᵀ demonstrated a broader substrate utilization profile, traits shared only in part by other relatives. More details on substrate utilization are provided in the species description below. Unlike most strains compared, meth-B3ᵀ uniquely utilized crystalline cellulose and exhibited weak growth on myo-inositol. Furthermore, it could metabolize glycerol and mannitol, in contrast to most of its close relatives. Growth on complex polysaccharides such as crystalline cellulose, xylan and starch was also positive, particularly distinguishing meth-B3ᵀ from species with more limited saccharolytic capabilities. Vitamins were not required for the growth of strain meth-B3ᵀ.

**Table 1.** Differential eco-physiological characteristics and genomic features that discriminate strain meth-B3^T^ from the most closely related type strains validly published within the order *Eubacteriales* Strains: 1, Strain meth-B3^T^ (present study); 2, *A. hokkaidonensis* R-7^T^ [23]; 3, *A. lactis* WTE2008^T^ [23]; 4, *C. minuta* YIT 12065^T^ [75]; 5, *C. hongkongensis* HKU16^T^ [56,57]; 6, *C. intestinihominis* AF73-05CM02^T^ [24]; 7, *C. tenuis* NSJ-35^T^ [15]; 8*, L. tenuis* NSJ-44^T^ [15]; 9, *G. tenuis* NSJ-53^T^ [15]; 10, *G. tenuis* NSJ-63^T^ [15].

Characteristics	1	2	3	4	5	6	7	8	9	10
Habitat	Landfill lab–scale methanogenic bioreactor	Sheep rumen	Cow rumen	Human faeces	Human blood	Human faeces	Human faeces	Human faeces	Human faeces	Human faeces
Gram-stain	–	–	–	+/–*	+	–	na	na	na	na
Cell shape	Rods	Rods	Rods	Rods	Coccobacilli/short rods	Rods	Rods	Ovoid	Ovoid/rods	Spherical
Cell size (µm)	0.75–1.08×1.50–2.08	1.0–3.0×0.2–0.5	1.0–3.0×0.2–0.5	0.8–1.9×0.4	0.7–1.1×0.4–0.5	1.0–2.0×0.5	1.2–1.6×0.6–0.8	1.3–1.6×0.7–0.9	1.3–2.0×0.7–0.9	0.8–1.1×0.8–1.1
Spore formation	–	–	–	–	–	–	–	–	–	–
Temperature range for growth (°C)	25–37 (35)	30–45	30–45	25–43 (37)	37	30–45 (37–42)	37	37	37	37
Salinity range for growth (%)	0–0.8 (0.7)	0.5	0.5	0–5	na	0–2	na	na	na	na
pH range for growth	6.5–8 (7)	5.6–7.0	5.9–7.0	6.0–9.0 (7.5)	na	6–8.5 (6.5–7.0)	na	na	na	na
Motility	+	–	–	–	+	–	–	–	–	+
**Genomic features†**										
DNA G+C content (mol%)	62.65	53.04	53.5	51.44	48.51	52.07	48.9	57.64	56.48	53.27
ANI (%)	100	65.35	65.77	63.77	63.03	63.64	62.84	66.21	64.83	64.27
AAI (%)	100	52.03	52.49	50.86	50.48	50.72	50.71	52.42	51.9	51.26
POCP (%)	100	28.11	26.97	28.54	28.7	29.12	27.72	31.25	29.55	30.08
**Growth on soluble substrates‡**										
Arabinose	+	+	+	+	+	+	–	–	–	–
Fructose	–	–	+	na	na	+	+	+	–	+
Galactose	+	+	(+)	na	na	+	–	+	–	+
Glucose	+	+	+	+	+	+	(+)	+	–	+
Cellobiose	+	+	+	–	–	–	–	+	–	–
Lactose	+	+	+	–	–	–	(+)	(+)	–	–
Maltose	+	+	+	–	–	(+)	–	+	–	–
Sucrose	+	+	+	–	–	+	+	–	+	–
Raffinose	+	(+)	(+)	–	–	(+)	–	–	–	–
Glycerol	+	–	–	–	+	–	–	(+)	(+)	+
Myo-inositol	(+)	–	–	na	na	–	–	(+)	–	–
Mannitol	+	–	–	–	–	–	–	(+)	–	–
**Growth on insoluble substrates§**										
Crystalline cellulose	+	–	–	na	na	na	na	na	na	na
Starch	+	+	+	na	na	–	na	na	na	na
Xylan	+	+	+	na	na	na	na	na	na	na
**Fermentation end products||**	L, A, P, B, V, E	A, L, H, E	A, L, H, E	A, B**	na	A, F, B, L**	na	na	na	na
**Major (>5 %) cellular fatty acids††**	C_15:0_ anteiso (36.46%), C_15:0_ iso (21.27%), C_14:00_ (15.28%), C_14:0_ iso (15.12%), C_16:00_ (5.85%)	C_17 : 0_ anteiso (19.7 %), C_16 : 0_iso (18.2 %), C_16 : 0_ (11.5 %), C_18 : 0_ (9.1%), C_15 : 0_ anteiso (8.9 %), C_18 : 0_ iso (6.6 %)	C_18 : 0_ (19.3 %), C_16 : 0_ (15.2 %), C_17 : 0_ anteiso (8.7 %), C_16 : 0_iso (8.4 %), C_15 : 0_ anteiso (7.7 %)	C_15 : 0_ iso (37.8 %), C_16 : 0_ (31.7 %), C_14 : 0_ (14.8 %)	na	C_14 : 0_ (46.6 %), C_16 : 0_ (9.7 %), C_10 : 0_ (7.5 %), C_15 : 0_ iso (7.4 %), C_12 : 0_ (7.2 %), C_18 :1_ω9*c* (6.9 %), C_11: 0_ iso (5.6 %)	na	na	na	na

*Described originally as Gram-negative [23,58], but later reports have shown Gram-positive staining of cells [56,58].

†Calculated in the present study directly from genomes.

‡Growth on soluble substrates was determined in basal medium (20 mM substrate) under the conditions described in the text; data for reference strains were compiled from the original species descriptions.

§Growth was assessed by fermentation end product formation. +, Production of >1 mM ethanol.

||A, acetate; B, butyrate; E, ethanol; F, formate; H, hydrogen; L, lactate; P, propionate; V, valerate.

**These studies do not report testing for the production of alcohols.

††Listed in order of abundance, with abundances (%) in brackets beside each cellular fatty acid. Full profiles are shown in Table 2.

Optimum values are given in parentheses. −, Negative; +, positive; (+), weak growth or result; na, data not available.

To further assess its ability to degrade complex lignocellulose biomass, functional annotation, classification and analysis of each gene in the newly sequenced genome of strain meth-B3^T^ were performed using COGclassifier 1.0.5 [59]. Additionally, carbohydrate-active enzymes (CAZy) analysis and glycoside hydrolase (GH) annotation were investigated using dbCAN3 2.0.0 [60] to explore the repertoire of CAZy and their respective predicted substrates. Genome analysis of strain meth-B3ᵀ revealed an extensive repertoire of carbohydrate-active enzymes (CAZymes) associated with lignocellulose degradation, suggesting a key ecological role in biopolymer turnover within anaerobic landfill environments (Fig. S1). The strain encodes numerous GHs, including GH13, GH94, GH3, GH8, GH25, GH2 and GH78, alongside carbohydrate-binding modules (CBMs) such as CBM16, CBM34, CBM48 and CBM67. These enzyme families are implicated in the hydrolysis of polysaccharides abundant in plant biomass, such as cellulose, hemicellulose (e.g. xylan, arabinan, glucomannan), starch and beta-glucan, as well as soluble carbohydrates such as sucrose [61,62]. Notably, families GH94 and GH8 are directly linked to cellulose metabolism [63,65], while GH13, GH77 and CBM48 target starch and glycogen [66,68]; GH3 and GH78 contribute to hemicellulose and arabinose side-chain cleavage [69]. The predicted substrates targeted by these CAZymes overlap substantially with the carbohydrate composition of maize residues [70,73]. Furthermore, COG category assignments indicate enrichment in genes related to carbohydrate transport and metabolism, a common genomic trait of efficient saccharolytic bacteria [74,75]. These genomic features support the role of strain meth-B3ᵀ in anaerobic degradation of maize-derived compounds, enhancing its potential relevance for bioconversion and biogas production in engineered or natural methanogenic ecosystems.

The strain was also tested for its ability to use various electron acceptors, including nitrate (10 mM), nitrite (5 mM), fumarate (10 mM) and oxygen. None of these electron acceptors supported or enhanced the growth of strain meth-B3ᵀ. Additional biochemical characterizations were performed using Analytical Profile Indexes API (20E and API 20NE systems; bioMérieux) under anoxic conditions. Consistent with the utilization of glucose, arabinose, mannitol, maltose, sucrose and raffinose, the API assays confirmed positive reactions for the fermentation or assimilation of these carbohydrates. Positive results were also observed for the hydrolysis of urea and the assimilation of citrate and formate, in agreement with growth on urea and citrate. These findings reinforce the metabolic versatility of strain meth-B3ᵀ, supporting its distinct phenotypic profile. Strain meth-B3^T^ was catalase negative and oxidase negative. The production of hydrogen sulphide was negative.

Volatile fatty acids, sugars and alcohols produced during fermentation of glucose were quantified using HPLC (High-performance Liquid Chromatography) on a Shimadzu LC-2030C 3D Plus system. Analyses were conducted using an Aminex HPX-87H column (300×7.8 mm, 9 µm particle size), with 0.005 N sulphuric acid as the mobile phase at a flow rate of 0.6 ml min^−1^. The column was maintained at 35 °C for a total runtime of 60 min. Detection of organic acids was performed via a UV detector set at 210 nm, while alcohols were monitored using a refractive index detector. All instruments were interfaced with LabSolutions software (version 5.92) for data acquisition and analysis. Culture supernatants were prepared by centrifugation (10,000***g***, 5 min), and 600 µl of the clarified supernatant was transferred to the autosampler. An injection volume of 20 µl was used per run. End-product analyses were performed using the defined basal medium supplemented with glucose (20 mM). The fermentation profile of glucose by strain meth-B3ᵀ demonstrated a mixed-acid pattern, with lactic acid, acetic acid, propanoic acid, butyric acid, valeric acid and ethanol detected as end products of glucose metabolism. In contrast, its close phylogenetic relative *C. intestinihominis* AF73-05CM02ᵀ has been reported to produce acetic, formic, butyric and lactic acids [24]. The presence of propanoic and valeric acids, as well as ethanol, in the metabolic profile of meth-B3ᵀ suggests distinct fermentative capabilities and indicates metabolic divergence from closely related taxa within the order *Eubacteriales*. Although detailed growth kinetics and end-product analyses of C₁ metabolism were not determined, these aspects warrant further physiological investigation to better understand the metabolism of strain meth-B3ᵀ.

The cellular fatty acid profile of strain meth-B3ᵀ was determined from cultures grown under the same conditions as those reported for *A. hokkaidonensis* R-7ᵀ and *A. lactis* WTE2008ᵀ [23], and analysed by the DSMZ-German Collection of Microorganism and Cell Cultures (Braunschweig, Germany) according to the standard protocol of the Sherlock Microbial Identification System (MIDI). The dominant fatty acids were C_15:0_ anteiso (36.46%), C_15:0_ iso (21.27%), C_14:0_ (15.28%) and C_14:0_ iso (15.12%), together constituting over 88% of the total fatty acids (Table 2). This composition distinctly differentiates meth-B3ᵀ from its closest phylogenomic relatives, *A. hokkaidonensis* R-7ᵀ and *A. lactis* WTE2008ᵀ. For example, while both *Aristaeella* strains showed notable amounts of C_17:0_ anteiso (up to 19.74%) and C_16:0_ iso, these fatty acids were either absent or significantly reduced in meth-B3ᵀ. Conversely, the prominent presence of C_15:0_ anteiso and C_14:0_ iso in meth-B3ᵀ was not mirrored in the *Aristaeella* strains. Additionally, meth-B3ᵀ lacked long-chain and unsaturated fatty acids such as C_18:0_ and C_18:1_ ω9c, commonly present in both *Aristaeella* taxa. These differences in fatty acid composition reinforce the phenotypic distinctiveness of meth-B3ᵀ and support its taxonomic separation at the genus and family levels.

**Table 2.** Whole-cell fatty acid profiles (percentage of total) of strain meth-B3^T^ and its closest relatives, *A. hokkaidonensis* R-7ᵀ and *A. lactis* WTE2008^T^

Cellular fatty acids	Strain meth-B3^T^	*A. hokkaidonensis* R-7ᵀ	*A. lactis* WTE2008^T^
C_10:00_	−	0.11	0.11
C_11:00_	−	−	0.05
C_11:0_ anteiso	−	0.05	−
C_12:00_	−	0.78	0.82
C_12:0_ iso	−	0.18	0.33
C_13:00_	−	0.2	0.38
C_13:0_ iso	0.72	0.26	0.48
C_13:0_ anteiso	−	0.42	0.94
C_13:0_ iso 3OH	−	0.13	0.19
C_14:00_	**15.28**	3.08	3.61
C_14:0_ iso	**15.12**	1.8	3.19
C_14:0_ DMA	−	−	0.19
C_15:00_	−	3.23	3.44
C_15:0_ iso	**21.27**	0.9	1.79
C_15:0_ anteiso	**36.46**	8.92	7.7
C_15:0_ iso DMA	−	0.43	0.67
C_15:0_ 3OH	−	0.17	0.67
C_16:00_	5.85	**11.54**	**15.17**
C_16:0_ iso	3.07	**18.19**	8.4
C_16:0_ 2OH	−	0.17	0.2
C_16:0_ 3OH	−	0.97	2.2
C_16:1_ ω7c	−	0.08	0.2
C_17:0_	−	4.3	2.99
C_17:0_ iso	−	0.48	0.7
C_17:0_ anteiso	−	**19.74**	8.72
C_17:0_ 3OH	−	0.44	0.95
C_17:0_ anteiso 3OH	−	0.78	2.39
C_18:0_	2.23	9.11	**19.32**
C_18:0_ iso	−	6.63	2.57
C_18:1_ ω6c	−	0.22	0.48
C_18:1_ ω9c	−	0.72	1.72
C_18:2_ ω6,9c	−	0.75	1.27
C_19:0_	−	0.5	0.35
C_19:0_ anteiso	−	1.85	0.78
C_20:0_	−	0.8	1.52
**Summed features**	−	2.07	5.5

−, Absent. Values >10 % are shown in bold type.

DMA, dimethyl acetal; 2OH, 3-hydroxy; 3OH, 3-hydroxy.

## Description of *Anaerozeibacter* gen. nov.

*Anaerozeibacter* (An.ae.ro.ze.i.bac’ter. Gr. pref. *an-* = not; Gr. masc. n. *aer*=air; L. fem. n. *Zea*, a genus of grasses including maize; N.L. masc. n. *bacter*=a rod; N.L. masc. n. *Anaerozeibacter*, an anaerobic rod-shaped bacterium associated with maize degradation).

Cells are strictly anaerobic, non-spore-forming, Gram-stain-negative rods. Cells are motile and display mesophilic and neutrophilic growth profiles. The genus is characterized by the ability to ferment a broad spectrum of sugars, amino acids and organic acids, including plant-derived polysaccharides such as cellulose and xylan. Metabolic end products include acetate, lactate, propionate, butyrate, valerate and ethanol. The major cellular fatty acids are C_15:0_ anteiso, C_15:0_ iso, C_14:0_ and C_14:0_ iso. The G+C content of the genomic DNA is around 62.6 mol%. Based on phylogenetic, chemotaxonomic and genome-based analyses, *Anaerozeibacter* represents a distinct lineage within the order *Eubacteriales*. The type species is *A. quisquiliarum*.

## Description of *Anaerozeibacter quisquiliarum* sp. nov.

*Anaerozeibacter quisquiliarum* (quis.qui.li.a’rum. L. gen. pl. n. *quisquiliae*, refuse or waste; N.L. gen. pl. n. *quisquiliarum*, ‘of refuse’, referring to the origin of the type strain isolated from landfill waste digesting maize-based biomass).

Cells are Gram-stain-negative, strictly anaerobic, non-spore-forming, rod-shaped and motile by means of polar flagella. Cell dimensions range from ~0.75 to 1.08 µm in width and ~1.50 to 2.08 µm in length. Colonies are small (1–2 mm in diameter), circular and warm-cream coloured after 7 days of incubation on modified FAB medium. The strain grows optimally at 35 °C (range, 25–37 °C), pH 7.0 (range, 6.5–8.0) and tolerates up to 0.8% NaCl (w/v), with optimal growth at 0.7%. Vitamins are not required for growth. Yeast extract is not essential but enhances growth.

In the presence of yeast extract, strain meth-B3ᵀ utilizes the following substrates: alanine, arabinose, arginine, asparagine, butyrate, cellobiose, citrate, cysteine, formate, galactose, glucose, glutamate, glutamine, glycerol, glycine, histidine, inositol, isoleucine, lactate, lactose, leucine, malate, maltose, mannitol, methanol, methionine, pyruvate, raffinose, succinate, sucrose, tryptophan, tyrosine and urea. No growth is observed on acetate, fructose, fumarate, gallic acid, gluconate, hippuric acid, methylamine or phenylalanine. For complex substrates, the strain was able to grow on yeast extract, tryptone, peptone, casamino acids, starch, xylan and cellulose, but not on gelatine or casein. Strain meth-B3ᵀ ferments glucose to produce lactate, acetate, butyrate, valerate and ethanol. No growth enhancement was observed in the presence of nitrate, nitrite or fumarate as electron acceptors.

The major cellular fatty acids are iso-C_15:0_, anteiso-C_15:0_, C_14:0_ and iso-C_14:0_. The DNA G+C content of the type strain is 62.65 mol%. The genome size is ~3.83 Mbp.

The type strain is meth-B3ᵀ (=DSM 112769ᵀ=ATCC TSD-269ᵀ), isolated from a laboratory-scale anaerobic methanogenic bioreactor digesting maize-based waste. The GenBank accession numbers for the 16S rRNA gene and genome are MW741711 and JBISDI000000000, respectively.

## Description of *Anaerozeibacteraceae* fam. nov.

*Anaerozeibacteraceae* (An.ae.ro.ze.i.bac.te.ra.ce’ae. N.L. masc. n. *Anaerozeibacter*, type genus of the family; –*aceae*, ending to denote a family; N.L. fem. pl. n. *Anaerozeibacteraceae*, the family whose nomenclatural type is the genus *Anaerozeibacter*).

The family is proposed based on phylogenetic analyses of 16S rRNA gene sequences and whole-genome comparisons. Cells are Gram-stain-negative, rod-shaped, obligately anaerobic and mesophilic. It belongs to the order *Eubacteriales* of the phylum *Bacillota*.

## Supplementary material

10.1099/ijsem.0.006985 Uncited Supplementary Material 1.

10.1099/ijsem.0.006985 Uncited Supplementary Material 2.

10.1099/ijsem.0.006985 Uncited Supplementary Material 3.

10.1099/ijsem.0.006985 Uncited Supplementary Material 4.

10.1099/ijsem.0.006985 Uncited Supplementary Material 5.

## Abbreviations

AAI: average amino acid identity
ANI: average nucleotide identity
APHA: Animal and Plant Health Agency
API: analytical profile index
CAZy: carbohydrate-active enZymes database
CAZymes: carbohydrate-active enzymes
CBM: carbohydrate-binding module
COG: Clusters of Orthologous Groups
dDDH: digital DNA–DNA hybridization
FAB: Fastidious Anaerobic Broth
GGDC: Genome-to-Genome Distance Calculator
GH: glycoside hydrolase
HCl: hydrochloric acid
HPLC: high-performance liquid cromatography
iTOL: Interactive Tree of Life
MIDI: Microbial Identification System
ML: maximum-likelihood
NaCl: sodium chloride
NaOH: sodium hydroxide
Na₂S·9H₂O: sodium sulphide nonahydrate
NCBI: National Center for Biotechnology Information
N₂/H₂/CO₂: nitrogen/hydrogen/carbon dioxide gas mixture
O₂: molecular oxygen
POCP: percentage of conserved protein
SLM: scale synthetic landfill microbiome
TEM: transmission electron microscopy
