# Supplementary material for: Anaerozeibacter quisquiliarum gen. nov., sp. nov., a novel mesophilic bacterium isolated from a laboratory-scale methanogenic landfill bioreactor digesting maize, and proposal of Anaerozeibacteraceae fam. nov., representing a new family within the order Eubacteriales
Source: Int J Syst Evol Microbiol. 2025 Dec 10;75(12):006985. doi: 10.1099/ijsem.0.006985 (PMC12694931; doi:10.1099/ijsem.0.006985)
Supplement: Uncited Supplementary Material 1. [file ijsem-75-06985-s001.pdf]

Supplementary Table 1. Estimates of Evolutionary Divergence of the 16S rRNA sequences between both strain meth-B3^{T} and strain meth-H3 sequences and their closely related species. Analyses were conducted using the Maximum Composite Likelihood model [1]. The rate variation among sites was modelled with a gamma distribution (shape parameter = 1). This analysis involved 36 nucleotide sequences. All ambiguous positions were removed for each sequence pair (pairwise deletion option). There were a total of 1454 positions in the final dataset. Evolutionary analyses were conducted in MEGA11 [2].

**Strains (GenBank accession no.)**	**Similarities (%)**	
	**Strain meth-B3^{T}**	**Strain meth-H3**
Anaerozeibacter quisquiliarum meth-B3^{T} (MW741711)	100.00	100.00
Anaerozeibacter quisquiliarum meth-H3 (PQ498816)	100.00	100.00
"Beduinella massiliensis" Marseille P2846^{T} (LT576387)	88.29	88.29
Aristaeella hokkaidonensis R-7^{T} (ON706269)	86.82	86.82
Aristaeella lactis WTE2008^{T} (ON706274)	86.80	86.80
Luoshenia tenuis NSJ-44^{T} (MT905125)	84.86	84.86
Christensenella intestinihominis AF73-05CM02^{T} (KX078376)	83.91	83.91
Gehongia tenuis NSJ-53^{T} (MT905128)	83.74	83.74
Christensenella tenuis NSJ-35^{T} (MT905171)	83.30	83.30
"Maliibacterium massiliense" Marseille P3954^{T} (LT985391)	82.69	82.69
"Beduinibacterium massiliense" Marseille P3337^{T} (LT631514)	82.44	82.44
Christensenella minuta YIT 12065^{T} (AB490809)	82.40	82.40
"Christensenella timonensis" Marseille P2437^{T} (LT223568)	81.80	81.80
"Christensenella massiliensis" Marseille P2438^{T} (LT161898)	81.33	81.33
Christensenella hongkongensis JCM 17853^{T} (AB671763)	80.75	80.75
Xylanivirga thermophila SYSU GA17129^{T} (MK170162)	80.47	80.47
Lutispora thermophila EBR46^{T} (AB186360)	80.30	80.30
Petroclostridium xylanilyticum SK-Y3^{T} (KT630605)	80.24	80.24
Guopingia tenuis NSJ-63^{T} (MT905129)	80.05	80.05
Clostridium pascui DSM 10365^{T} (X96736)	79.47	79.47
Caldicoprobacter guelmensis D2C22^{T} (JQ707908)	79.45	79.45
Gracilibacter thermotolerans JW/YJL-S1^{T} (DQ117466)	79.32	79.32
Clostridium tetanomorphum DSM 4474^{T} (DQ241819)	79.26	79.26
Caldicoprobacter algeriensis TH7C1^{T} (GU216701)	79.24	79.24
Caldicoprobacter oshimai JW/HY-331^{T} (AB450762)	79.14	79.14
Caldicoprobacter faecalis DSM 20678^{T} (FR749980)	78.94	78.94
Mahella australiensis 50-1 BON^{T} (AY331143)	78.80	78.80
Clostridium lundense DSM 17049^{T} (AY858804)	78.74	78.74
Acetivibrio thermocellus JCM 9322^{T} (AB558166)	78.45	78.45
Lutispora saccharofermentans m25^{T} (MW962255)	78.39	78.39
Acetivibrio cellulolyticus ATCC 33288^{T} (L35516)	77.72	77.72
Acetivibrio cellulosolvens ATCC 35928^{T} (L35515	77.70	77.70
Acetivibrio clariflavus EBR45^{T} (AB186359))	77.12	77.12
Acetivibrio aldrichii DSM 6159^{T} (X71846)	76.60	76.60
Acetivibrio alkalicellulosi Z-7026^{T} (AY959944)	75.50	75.50
Desulfobulbus oligotrophicus Prop6^{T} (NR_156089)	69.27	69.27

1. Tamura K., Nei M., and Kumar S. (2004). Prospects for inferring very large phylogenies by using the neighbor-joining method. Proceedings of the National Academy of Sciences (USA) 101:11030-11035.

2. Tamura K., Stecher G., and Kumar S. (2021). MEGA 11: Molecular Evolutionary Genetics Analysis Version 11. Molecular Biology and Evolution https://doi.org/10.1093/molbev/msab120.

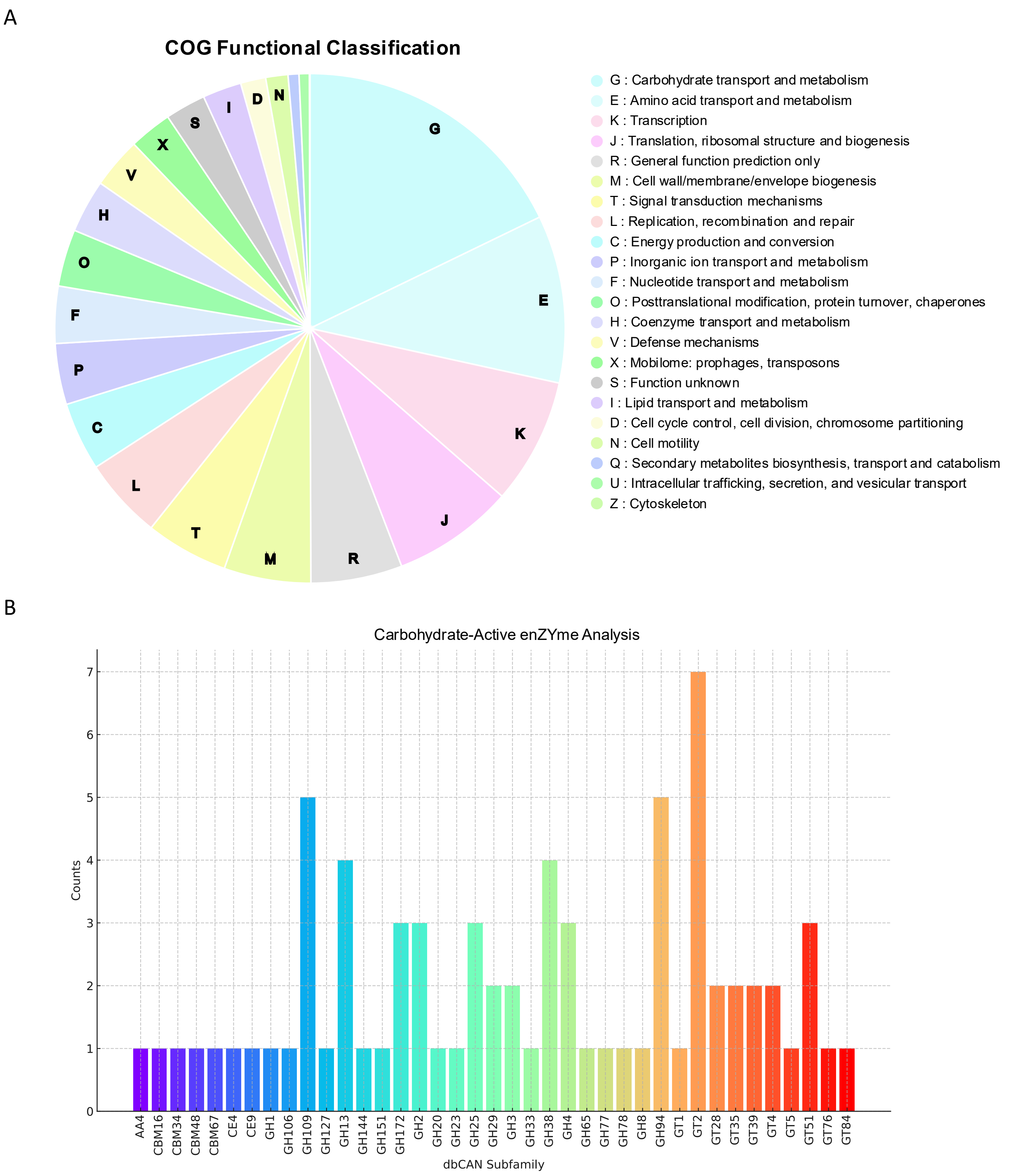

Fig. S1. A) The distribution of genes associated with COG functional categories in strain meth-B3^{T}. B) Carbohydrate-Active enZYme Analysis of strain meth-B3^{T}. CBMs, Carbohydrate-Binding Modules; CEs, Carbohydrate Esterases; GHs, Glycoside Hydrolases; GTs, Glycosyl Transferases; PLs, Polysaccharide Lyases.
